# Tonsil-derived mesenchymal stem cell-embedded *in situ* crosslinkable gelatin hydrogel therapy recovers postmenopausal osteoporosis through bone regeneration

**DOI:** 10.1371/journal.pone.0200111

**Published:** 2018-07-05

**Authors:** Gyungah Kim, Yoon Shin Park, Yunki Lee, Yoon Mi Jin, Da Hyeon Choi, Kyung-Ha Ryu, Yoon Jeong Park, Ki Dong Park, Inho Jo

**Affiliations:** 1 Department of Molecular Medicine, College of Medicine, Ewha Womans University, Seoul, Republic of Korea; 2 Ewha Tonsil-derived mesenchymal Stem cells Research Center (ETSRC), College of Medicine, Ewha Womans University, Seoul, Republic of Korea; 3 Major in Microbiology, School of Biological Sciences, College of Natural Sciences, Chungbuk National University, Cheongju, Republic of Korea; 4 Department of Molecular Science and Technology, Ajou University, Suwon, Republic of Korea; 5 Department of Pediatrics, College of Medicine, Ewha Womans University, Seoul, Republic of Korea; 6 Department of Dental Regenerative Biotechnology, Dental Research Institute, School of Dentistry, Seoul National University, Seoul, Republic of Korea; 7 Central Research Institute, Nano Intelligent Biomedical Engineering Corporation (NIBEC), Seoul, Republic of Korea; Nanjing Medical University, CHINA

## Abstract

We investigated therapeutic potential of human tonsil-derived mesenchymal stem cells (TMSC) subcutaneously delivered to ovariectomized (OVX) mice for developing more safe and effective therapy for osteoporosis. TMSC were isolated from tonsil tissues of children undergoing tonsillectomy, and TMSC-embedded *in situ* crosslinkable gelatin-hydroxyphenyl propionic acid hydrogel (TMSC-GHH) or TMSC alone were delivered subcutaneously to the dorsa of OVX mice. After 3 months, three-dimensionally reconstructed micro-computed tomographic images revealed better recovery of the femoral heads in OVX mice treated with TMSC-GHH. Serum osteocalcin and alkaline phosphatase were also recovered, indicating bone formation only in TMSC-GHH-treated mice, and absence in hypercalcemia or other severe macroscopic deformities showed biocompatibility of TMSC-GHH. Additionally, visceral fat reduction effects by TMSC-GHH further supported their therapeutic potential. TMSC provided therapeutic benefits toward osteoporosis only when embedded in GHH, and showed potential as a supplement or alternative to current therapies.

## Introduction

Osteoporosis, a disease in which bones become fragile and very susceptible to breaking, is prevalent among older individuals, especially postmenopausal women [1]. Although the weakening of the bone may not have serious effects *per se*, the resulting bone fractures can be painful, inconvenient, damaging, and even fatal [2,3]. In the elderly, healing is stagnant and the immobility that follows a fracture often leads to a permanent loss of vitality. The drugs currently available to treat osteoporosis are largely antiresorptives that stall the breakdown of bone; bisphosphonates are often used as a first-line therapy, and hormone replacement therapy (HRT) is occasionally used for postmenopausal osteoporosis. Parathyroid hormone (PTH) may be used as an osteoanabolic. However, patients using these medications suffer from side effects. For example, the widely used formulation of oral bisphosphonate must be taken early in the morning, before breakfast, and the patient must thereafter maintain a supine position and an empty stomach for 30–60 min to prevent esophageal ulcers [3]. There are also far more serious adverse effects, such as bisphosphonate-related osteonecrosis of the jaw, thromboembolic complications from taking HRT and hypercalcemia from taking PTH [1,2]. Furthermore, long-term use of bisphosphonate may be associated with additional femoral fractures [4].

Stem cell therapy holds promise for patients suffering from organ dysfunction. Especially, adult stem cells such as mesenchymal stem cells (MSC) do not have safety issues of cancerous transformation or immune rejection and are currently utilized in the clinic for various purposes. Establishing new stem cell source is essential for further development of MSC therapy. Our group previously reported that discarded tonsillar tissues can be a new source of tonsil-derived MSC (TMSC), which express MSC surface antigens and show trilineage mesodermal differentiation as well as immunosuppression characteristic to MSC [5]. Their expressions of MSC-specific surface markers and embryonic stem cell markers are not altered up to at least passage 15 [6]. They are highly proliferative (doubling time of 37.1 ± 3.4 h), more so than the bone marrow counterpart (doubling time of 58.2 ± 2.3 h) [7]. TMSC are suited to tissue engineering [8], and useful against various conditions, such as liver fibrosis [9,10], diabetes mellitus [11], allergic rhinitis [12], tenocyte regeneration [13], Charcot-Marie-Tooth disease [14] and hypoparathyroidism [15–17].

As evidence continues to emerge regarding the relationship between decreasing MSC function and osteoporosis [18–20], MSC therapy is becoming an increasingly attractive alternative or additive for the current osteoporosis treatments [21]. To date, there have been attempts to treat osteoporosis with MSC therapy in animal models [22,23]. The previous studies mostly chose intra-bone marrow (IBM) or intra-tail venous injections as the injection route [24]. Our recent study also injected TMSC into the tail vein of senescence-accelerated mouse prone 6 mice [20]. However, systemically delivered MSC interfere with the circulation [25]and the immune functions [26], and the same risks exist for IBM route because the bone is an organ rich in blood vessels. Here, we sought to expand upon the prior studies by employing subcutaneous injection, which is safe and convenient.

Subcutaneous injection usually results in cell loss. There have long been issues with the survival and retention of stem cells delivered directly to the site of injury, and it has been suggested that a scaffold carrier should be used to support cell viability and function [27]. Our previous study in parathyroidectomized (PTX) model mice supported this perspective [17]. Scaffold can prevent cell death and promote cell function by supporting the efficient transport of food, waste products, and small molecules required for healing [28]. Several previous studies showed that the use of scaffold can boost the therapeutic effects of stem cells. For example, injections of TMSC embedded in Matrigel, but not TMSC alone, increased the survival rates of PTX rats [17]. A recent study showed TMSC delivered with Matrigel promote bone regeneration in an osteoradionecrosis rat model [29]. Furthermore, TMSC that had been three-dimensionally (3D)-cultured in poly(ethylene glycol)-poly(L-alanine-co-L-phenyl alanine) thermogel showed better chondrogenic differentiation [30], and TMSC in well-defined mesocrystals (4–8 μm) of calcium phosphate and polypeptide thermogel induced improved osteogenesis [31]. Among the various scaffold substances tested to date, we selected gelatin-based gel for the present study because of its outstanding biocompatibility, biodegradability, and non-immunogenicity [32]. Hydrogel is a jelly-like biomaterial consists of 3-dimensional networks of hydrophilic polymers, and gelatin-based hydrogel is one type of hydrogel [33,34]. The utilized gelatin gel can be made *in situ* by horseradish peroxidase (HRP)-catalyzed crosslinking, and was previously shown to be excellent for cell delivery [32]. Moreover, this gel may be easily manipulated in terms of its physicochemical properties (*e*.*g*., gelation time, matrix strength, and degradation rate), which offers controllability (*e*.*g*., to allow users to fine-tune cell function) [32,35].

Here, we utilized a gelatin-hydroxyphenyl propionic acid (GHPA) hydrogel (GHH) scaffold, which offers the benefits of biocompatibility, non-immunogenicity, and controllability [32]. Our present results clearly show that TMSC-embedded gelatin hydrogel (TMSC-GHH), but not TMSC alone, induced recovery in the ovariectomized (OVX) mouse model of osteoporosis.

## Materials and methods

### Materials

Type A gelatin from porcine skin, 1-ethyl-3-(3-dimethylaminopropyl)-carbodiimide (EDC), *N*-hydroxysuccinimide (NHS), 3-(4-hydroxyphenyl) propionic acid (HPA), hydrogen peroxide (H_2_O_2_; 30 wt% in H_2_O), HRP, ascorbic acid, β-glycerophosphate, dexamethasone, 17β-estradiol, and bovine serum albumin, and DNase were purchased from Sigma-Aldrich (St. Louis, MO, USA). Dimethylformamide (DMF) was purchased from Junsei Chemical, Co., Ltd. (Tokyo, Japan). Collagenase type I and the LIVE/DEAD^®^ Cell Assay kit were purchased from Invitrogen (Thermo Fisher Scientific, Waltham, MA, USA) and Ficoll-Paque was purchased from GE Healthcare (Little Chalfont, UK). High-glucose (4500 mg/L) Dulbecco’s Modified Eagle Medium (DMEM-HG) and Dulbecco’s phosphate buffered saline (DPBS) were purchased from Welgene Inc. (Gyeongsan, Korea). Trypsin-ethylenediamine tetra acetic acid, fetal bovine serum (FBS), penicillin-streptomycin, and antibiotic-antimycotic were purchased from GibcoBRL (Thermo Fisher Scientific, Waltham, MA, USA). Zoletil and Rompun were purchased from Virbac (Carros, France) and Bayer AG (Leverkusen, Germany) respectively. Serum separator tubes were purchased from Greiner Bio-One GmbH (Kremsmünster, Austria), and the osteocalcin (OCN) enzyme-linked immunosorbent assay (ELISA) kit was purchased from Immunotopics, Inc. (Quidel Corporation, Athens, OH, USA). Paraformaldehyde in phosphate buffered saline (PBS) (4% w/v) was obtained from Biosesang Co., Ltd. (Seongnam, Korea). All of the utilized chemicals were of analytical grade.

### TMSC isolation and culture

TMSC were isolated and cultured as previously described [17,36]. Discarded tonsils were obtained from children who underwent tonsillectomy at the Department of Otorhinolaryngology–Head and Neck Surgery, Ewha Womans University Medical Center (EWUMC, Seoul, Korea). We obtained informed written consent from parents or guardians of those who participated in the study.

The study protocol was approved by the institutional review board of Ewha Womans University Medical Center (EWUMC, IRB No. ECT-11-53-02). Isolated tonsillar tissues were mechanically minced and then enzymatically digested with collagenase type I and DNase at 37 °C for 30 min. Subsequent filtration and Ficoll-Paque density gradient centrifugation were performed to obtain adherent mononuclear cells, which were cultured in DMEM-HG supplemented with 10% FBS. After 48 h, the non-adherent cells were discarded, and the adherent mononuclear cells (hereafter called TMSC) were cultured. All TMSC used in this study were of passages 5 through 7.

### Synthesis of phenol-conjugated gelatin polymer

We synthesized GHPA as described previously [35]. Briefly, HPA was activated with EDC and NHS in a co-solvent of 3:2 (volume ratio) deionized water and DMF, and the activated HPA solution was stirred with pre-heated gelatin solution at 40 °C for 24 h. Purification with a dialysis bag (molecular weight cut-off = 3.5 kDa), followed by lyophilization, was used to obtain the GHPA polymer. The chemical structure of the GHPA conjugate was characterized by ^1^H NMR and ultraviolet spectrophotometry (V-750; Jasco, Tokyo, Japan).

### *In situ* formation of GHH and incorporation of TMSC for injection

GHH was prepared as previously described [27]. Briefly, GHPA/HRP solution (150 μL) was prepared by mixing GHPA and HRP, while GHPA/H_2_O_2_ solution (150 μL) was separately prepared by mixing GHPA and H_2_O_2_. Immediately prior to injection, the solutions were combined to make 300 μL of GHH with a strength of 4.4 kPa. The final concentrations of HRP and H_2_O_2_ were 0.005 mg/mL and 0.0075 wt%, respectively. To incorporate TMSC into the GHH matrix, 1×10^5^ TMSC were added to the GHPA/HRP solution before it was mixed with the GHPA/H_2_O_2_ solution.

### *In vitro* viability assay of TMSC-embedded GHH

The viability of TMSC embedded in GHH was measured using LIVE/DEAD^®^ assay reagents (50 mM calcein-acetoxymethyl ester and 25 mg/mL ethidium homodimer-1) in culture medium for 40 min at 37 °C. The labeled cells were observed and images were acquired at the indicated time points (0, 3, 7, 10, 14, and 21 d after plating) at 100× magnification using a FV300 confocal microscope (Olympus, Tokyo, Japan). Fluorescence signals were quantified and expressed in arbitrary units (a.u.).

### Generating the mouse model of osteoporosis

Fifty female Institute of Cancer Research (ICR) mice (weights between 18‒20 g) that had been OVX at 8 weeks of age as previously described [37] were obtained from KNOTUS Co., Ltd. (Guri, Korea). Upon arrival, the OVX mice (average weight at this point = 32.4 ± 2.44 g) were randomly assigned to following groups: Untreated (n = 10), Estrogen (n = 10), TMSC injection once (TMSC/1×; n = 5), TMSC injection twice (TMSC/2×; n = 5), TMSC-GHH injection once (TMSC-GHH/1×; n = 10), and TMSC-GHH injection twice (TMSC-GHH/2×; n = 10). As controls, we used five female ICR mice without OVX (non-OVX).

All mice were maintained in standard cages (five mice per cage) under constant temperature (22 ± 5 °C) and humidity (50 ± 10%), and with a 12-h light-dark cycle. All OVX mice were fed a calcium-deficient diet (80 mg calcium/kg diet), while the non-OVX mice were fed a standard chow diet; all mice were given *ad libitum* access to food and water. At 3 months after OVX, blood was collected from the jugular vein of each mouse into a serum separator tube. Each tube was centrifuged at 1300 ×g for 20 min, and the isolated serum was stored at -80 °C until use. The collected sera were subject to serum OCN measurement. All animal experiments were conducted in accordance with the guidelines of the Animal Ethics Committee at Ewha Womans University, by whom the experimental procedures were approved (ESM 12–0208).

### Treatment

For Estrogen mice, 17β-estradiol (1.0 mg/mouse) was intraperitoneally injected five times per week in a volume of 300 μL/injection. For the TMSC and TMSC-GHH groups, mice were first anesthetized by intraperitoneal injection with a mixture of Zoletil (0.25 mL/mouse) and Rompun (0.25 mL/mouse), and then 1×10^5^ cells/300 μL/mouse were injected subcutaneously in the back using a 22-G needle. The single-injection groups (TMSC/1× and TMSC-GHH/1×) were injected once at the beginning of treatment, while the double-injection groups (TMSC/2× and TMSC-GHH/2×) were injected a second time at 1.5 months after the initial injection. For untreated group, OVX mice received no treatment. All groups were followed for 3 months after the first treatment.

### Biochemical analysis of blood samples

To verify osteoporosis development and treatment efficacy, sera were collected at 3 months after OVX and at the end of the experiment. Collected sera were tested for serum OCN concentration using ELISA, for serum alkaline phosphatase (ALP) level using a Hitachi Clinical Analyzer 7180 (Hitachi Ltd., Tokyo, Japan), and for serum total calcium concentration using a 9180 Electrolyte Analyzer (Roche Diagnostics, Basel, Switzerland). The final serum concentrations were adjusted with respect to the initial concentrations.

### Micro-computed tomographic (microCT) analysis

MicroCT images of femoral heads were obtained using SkyScan 1172 microCT device (Bruker microCT, Kontich, Belgium). At the end of the experiment (3 months after the initial treatment), a hind limb containing a femoral head was isolated from each mouse, fixed in 4% paraformaldehyde, and stored at 4 °C until use. Femoral heads were scanned at 141 μA/70 kV for 590 ms through a 0.5-mm filter at a resolution of 4.84 μm. Images were reconstructed using the NRecon software (Bruker microCT), and cross-sectional images were obtained using the DataViewer software (Bruker microCT). BMD measurements and bone morphometric analyses were performed using the insides of the femoral heads, excluding the outermost 0.2-mm compact bone layers. BMD values were calibrated using the measurements obtained from a bone-density phantom of 0.25 g/cm^3^ and 0.75 g/cm^3^, and were calculated using the CTAn software (Bruker microCT). To generate 3D slice images of femoral heads, the middle 70 continuous sections of the analyzed femoral head regions were 3D-reconstructed using the CTVol software (Bruker microCT).

### Postmortem organ collection

At the end of the experiment, all remaining mice were sacrificed by CO_2_ euthanasia followed by cervical dislocation, following the guidelines of the Animal Ethics Committee at Ewha Womans University. For the subcutaneous cell-implanted groups, mice were first anesthetized with a 1:1 mixture of Zoletil/Rompun, their backs were cut open, and the injection sites and surrounding vascularization were examined. This was promptly followed by sacrifice. After euthanasia, periovarian and parametrial fat pads were collected for visceral fat mass measurements. Livers and kidneys were removed (without previous blood drainage) and weighed, and their macroscopic morphologies were examined.

### Statistical analysis

All values are presented as the mean ± standard deviation. All the data points from animals that died before the experimental endpoint were omitted. The presented graphs were generated using the GraphPad Prism 5 software (GraphPad Software Inc., La Jolla, CA, USA). Statistical significance was analyzed using the Student’s *t*-test. The level of significance is represented as *P < 0.05, **P < 0.01, or ***P < 0.001, and N.S. indicates absence of statistical significance.

## Results

### General characteristics of the animal model

**Fig 1:**
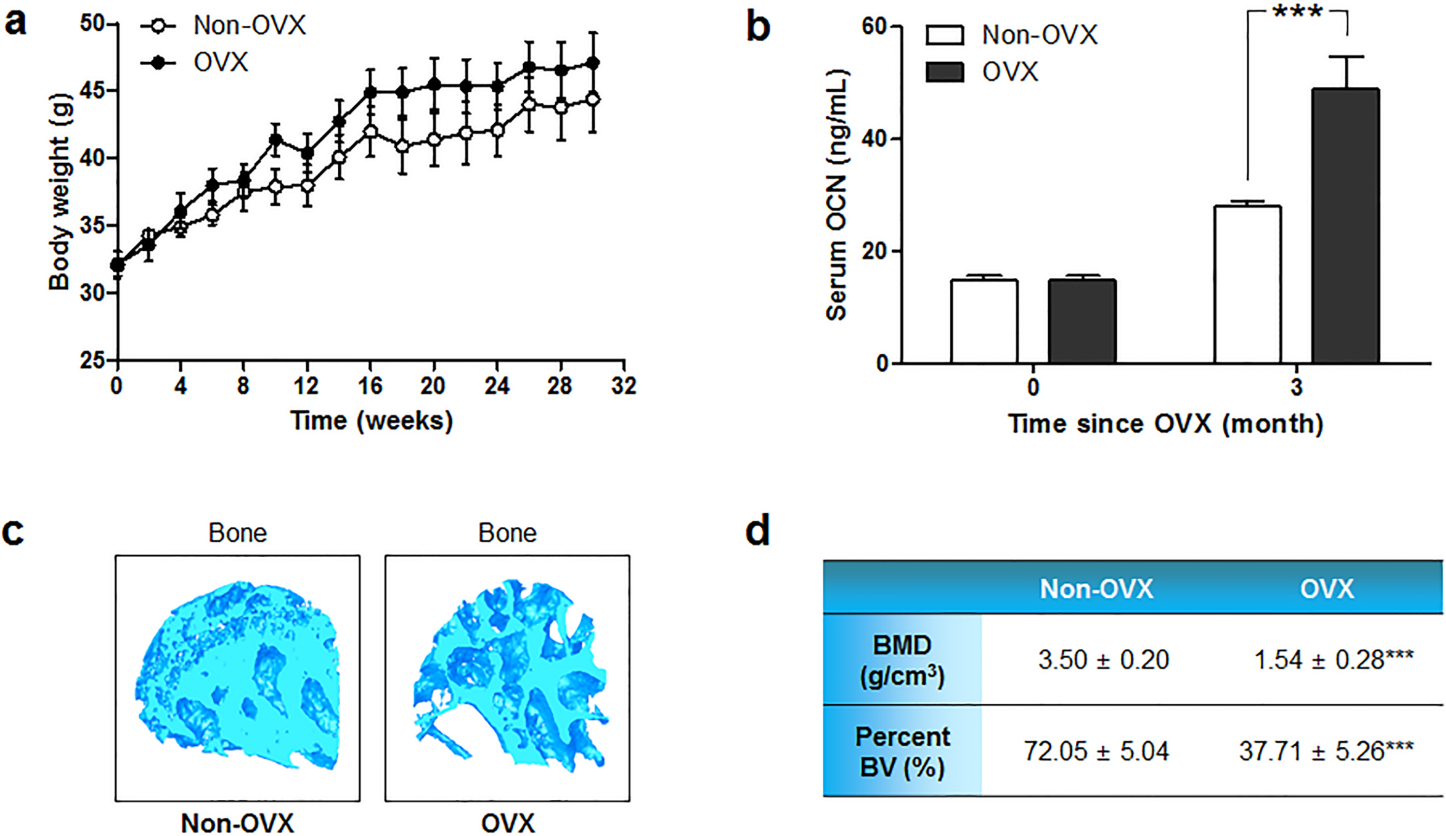
General characteristics of the OVX mouse model. (a) Body weight changes of OVX and non-OVX mice were traced from 8 weeks of age, when OVX was performed, to the end of the experimental period. (b) Serum OCN concentrations were measured in sera collected from non-OVX and OVX mice at 0 and 3 months after OVX. (c) 3D-constructed microCT images of the femoral head trabeculae of non-OVX and OVX mice at necropsy. (d) BMD and BV/TV values of the femoral head trabecular regions at necropsy from non-OVX and OVX mice, as analyzed from microCT images. Statistical significance is marked as ***P < 0.001.

To establish the osteoporosis mouse model, mice underwent bilateral OVX at 8 weeks of age to induce menopause-associated osteoporosis, and were fed low-calcium diet to accelerate the induction of osteoporosis. As a control, five non-OVX mice were subjected to no surgery or treatment, and were fed a standard chow diet throughout the experimental period. All mice had similar body weights at the starting point of therapies and throughout the experiment (Fig 1a).

We used the level of serum OCN, which is known to increase in osteoporotic patients [38], as a marker for osteoporosis. The mean initial OCN level was 14.8 ± 3.63 ng/mL, while that obtained at 3 months post-surgery was 48.9 ± 18.46 ng/mL (Fig 1b). This significant increase versus the baseline and non-OVX controls indicated that osteoporosis was successfully induced [37,39]. The serum OCN level of OVX mice was 1.75 times higher than that of non-OVX mice at 3 months post-surgery. Additionally, successful induction of osteoporosis was confirmed by microCT analysis of the hind limb at necropsy, which showed that the femoral head trabeculae of OVX mice were more porous and empty in architecture (Fig 1c) and had lower bone mineral density (BMD) and percent bone volume (bone volume/total volume; BV/TV) (Fig 1d).

### Preparation and injection of TMSC-loaded GHH

**Fig 2:**
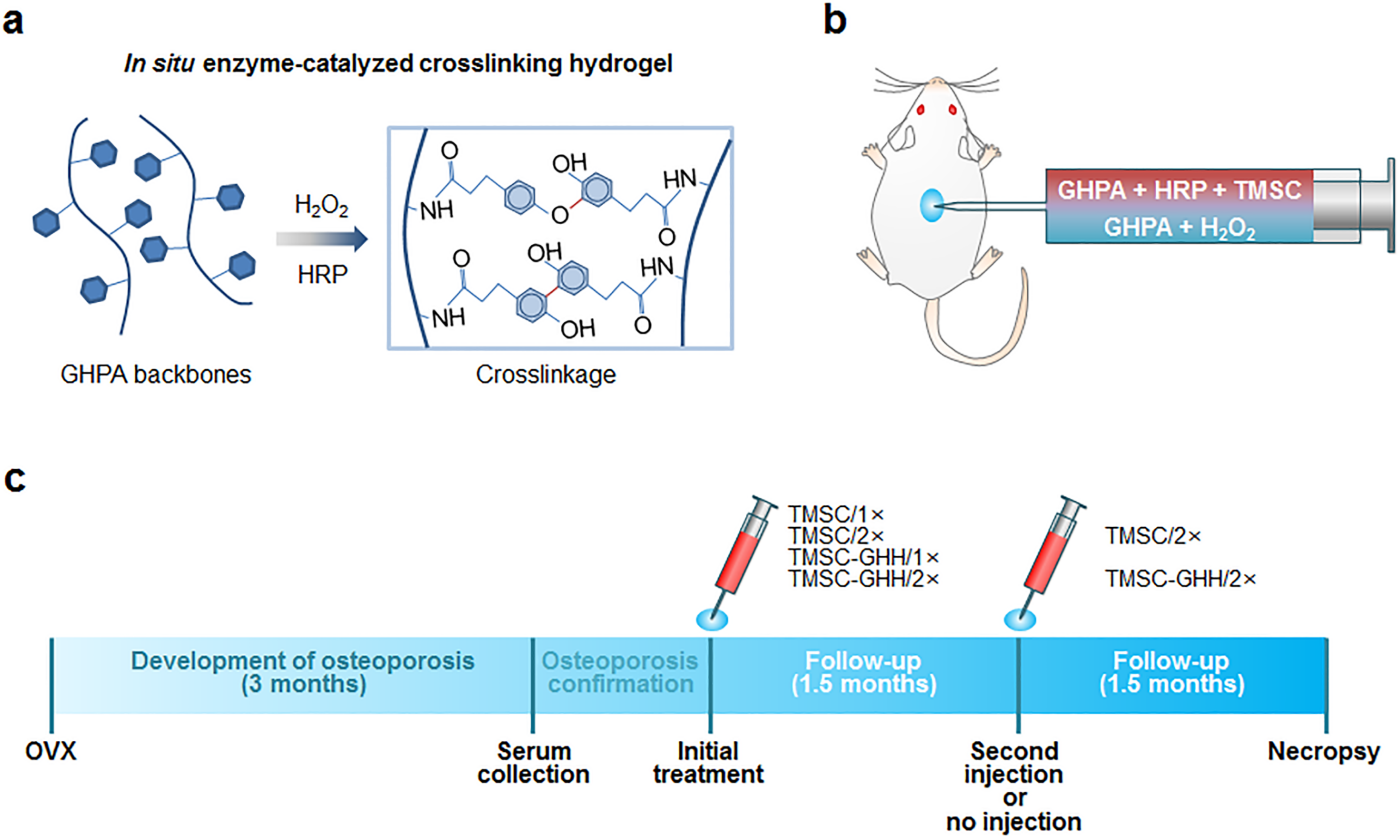
Schematic process used to prepare TMSC-GHH for injection, and overall experimental timeline. (a) GHPA polymers were enzymatically *in situ* crosslinked to form hydrogel, and TMSC were embedded in the hydrogel meshwork. (b) For TMSC-GHH injections, one solution containing GHPA polymers, HRP, and TMSC was mixed with another solution containing GHPA polymers and H_2_O_2_ at the time of injections. (c) Experimental procedures including the injection schedule and allotted durations.

We synthesized scaffold with gelatin-based GHH through in situ HRP-catalyzed crosslinking (Fig 2a), and loaded them with TMSC to enable us to inject mice with TMSC-GHH (Fig 2b). OVX mice were injected with TMSC alone or with TMSC-GHH once (1×) or twice (2×) during the 3-month period (Fig 2c).

**Fig 3:**
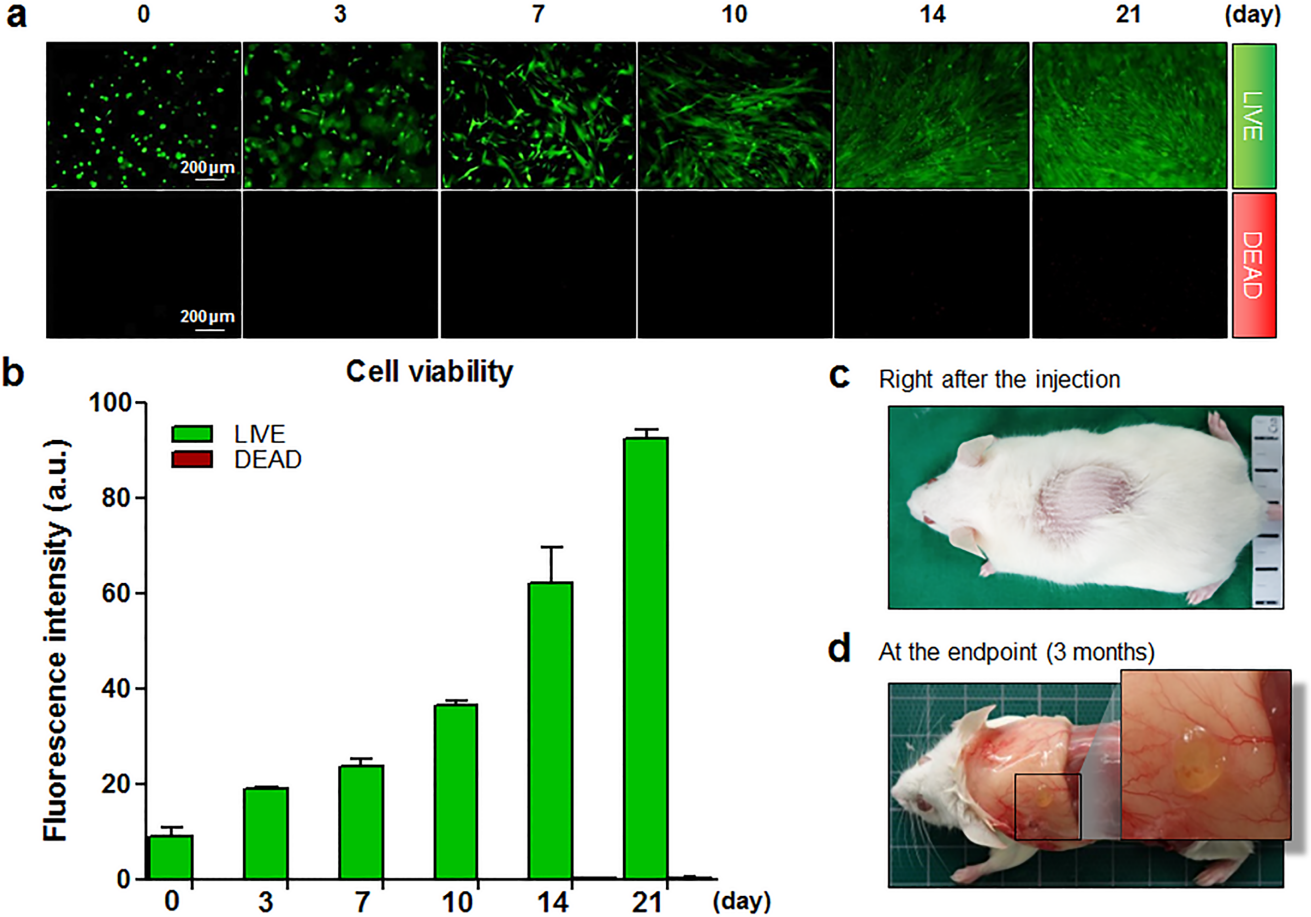
*In vitro* viability assay of TMSC-GHH over a 3-week period, and macroscopic morphology of TMSC-GHH implants. (a) TMSC-embedded GHH were plated on a 24-well plate, and LIVE/DEAD^®^ assays were performed at designated times. Horizontal sections of the entire depth of the TMSC-GHH were viewed under a microscope (×10 magnification). Live cells appear green (top row) and dead cells appear red (bottom row). (b) Fluorescence signals from LIVE/DEAD^®^ assays were quantified and expressed in arbitrary units (a.u.). (c) TMSC-GHH was injected subcutaneously in the dorsum. (d) Three months after treatment, TMSC-GHH-treated mice were anesthetized, and the subcutaneous layer of the dorsum was exposed to photograph the remaining hydrogel and nearby blood vessels.

LIVE/DEAD^®^ assays confirmed that almost all TMSC were highly viable and proliferative within the middle of each GHH, both qualitatively (Fig 3a) and quantitatively (Fig 3b). Maintenance of the spindle shape of TMSC and increased cell population suggest that GHH provided favorable growth environment. Especially as a component of extracellular matrix (ECM), gelatin supports cell functions such as cell adhesion, dispersion and proliferation through its direct interactions with the seeded cells [40]. GHH with its porosity has also been reported to allow thorough transport of nutrients and considerable vitality for cells. This remarkable biocompatibility of GHH was apparent in our results.

Fig 3c shows the areas where TMSC-GHH was implanted, and Fig 3d confirms that the gel formed successfully and retained their shapes for the 3-month experimental period. There was no trace of inflammation or necrosis near the implant site, suggesting that TMSC-GHH is biocompatible and may not cause immune rejection. Much more vascularization was detected in this region of TMSC-GHH-treated animals compared with those treated with PBS or TMSC alone (data not shown).

### TMSC-GHH recovers bone loss in OVX mice *in vivo*

**Fig 4:**
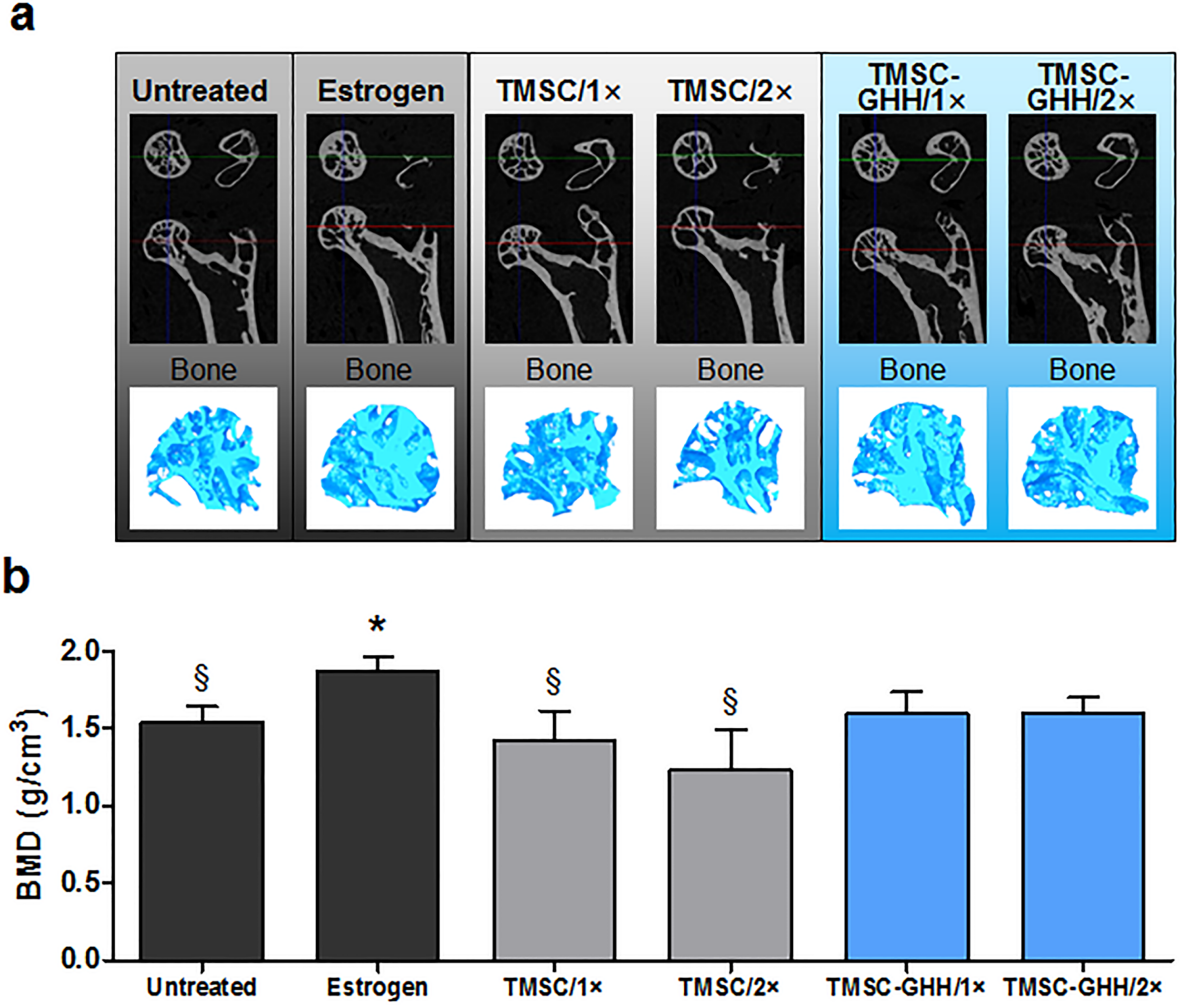
MicroCT images and BMD of femoral head trabeculae at the experimental endpoint. (a) Representative horizontal and coronal cross-sectional images capturing the proximal end of a femur as well as a 3D-constructed image of the femoral head trabecular bone for each group. (b) BMD values calculated from the microCT images of femoral head trabeculae. Statistical significance is marked as *P < 0.05 against Untreated group, and §*P* < 0.05 against Estrogen group.

To test the therapeutic effects of TMSC-GHH on osteoporosis, we isolated hind limbs from treated and untreated mice and performed microCT analysis. Fig 4a shows cross-sectional images of the proximal ends of the hind limbs, along with 3D-reconstructed images of femoral head trabeculae. As expected, the femoral heads of untreated mice had a porous structure, and estrogen treatment (positive control) recovered this defect. Treatment with TMSC-GHH, but not TMSC alone, appeared to implement some improvement of the bone structure by replenishing the femoral head trabeculae.

Compared with untreated control group, the Estrogen group also displayed a significant increase in BMD (Fig 4b). This increased BMD was not statistically different from those of TMSC-GHH groups, but was significantly higher than those of untreated and TMSC-only-treated mice. Based on improvement observed from trabecular images of the femoral head (Fig 4a), these findings suggest that the BMD elevation observed in TMSC-GHH-treated mice can be considered clinically significant. Notably, the BMD improvements did not differ between the TMSC-GHH/1× and TMSC-GHH/2× groups.

### TMSC-GHH treatment improves serum markers *in vivo*

**Fig 5:**
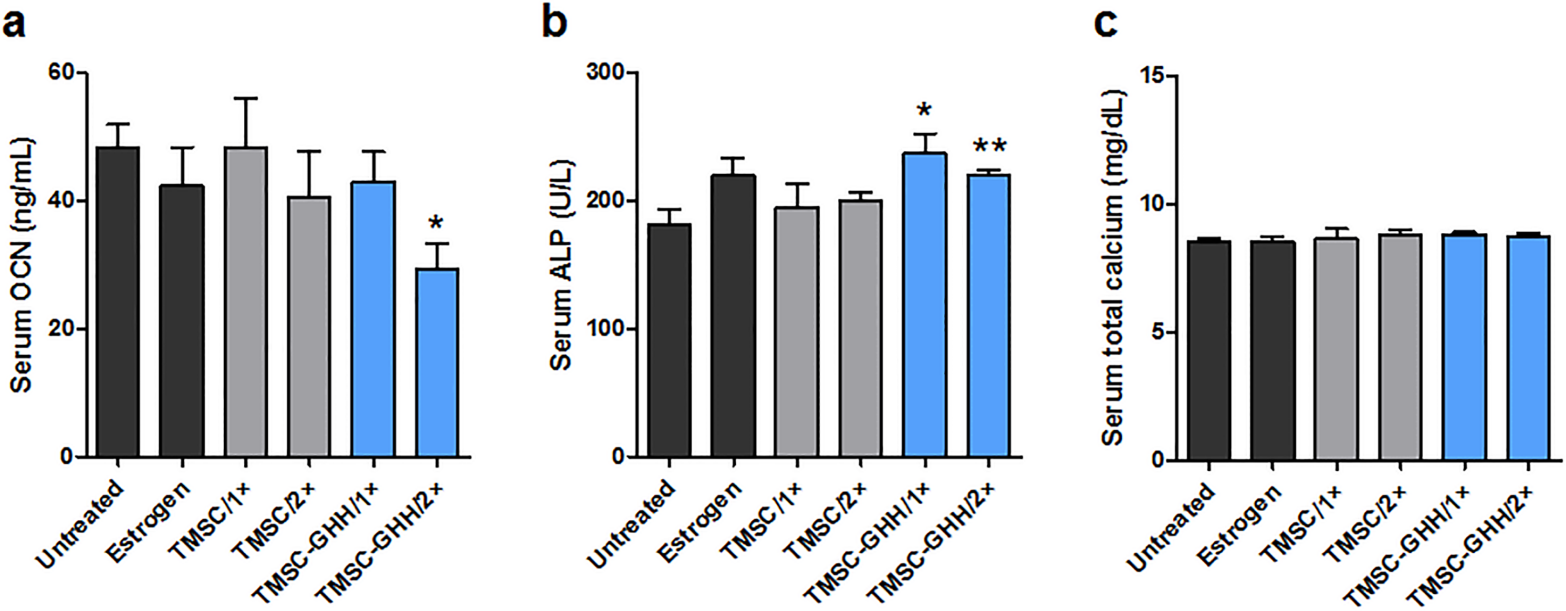
Serum OCN, ALP, and calcium levels of OVX mice. (a,b) Measurements of OCN and ALP levels from sera sampled at 3 months after first treatment. (c) The serum total calcium levels without adverse effects. Statistical significance is marked as *P<0.05 and **P<0.01.

Sera were collected from mice at 3 months after initial treatment and analyzed for the bone-related serum markers, OCN and ALP. The OCN levels were decreased in the Estrogen and TMSC-GHH groups relative to the untreated OVX mice (Fig 5a). In the beginning of the treatment, serum OCN concentration had been higher in our OVX model than in non-OVX mice (Fig 1b), which is consistent with the known increase of this marker in postmenopausal osteoporosis due to an imbalance in bone turnover. As increased OCN levels are known to be rescued upon therapeutic recovery of disturbed bone metabolism, our findings suggest that bone turnover was normalized in the Estrogen and TMSC-GHH groups. Interestingly, double injection of TMSC-GHH was particularly effective (even better than the Estrogen group) in suppressing the OVX-associated increase of OCN.

We also measured serum ALP concentration, which is a well-known bone formation marker. At month 3 post-treatment, relative to the untreated group, the Estrogen group showed a non-significant increase in ALP, whereas both the TMSC-GHH/1× and TMSC-GHH/2× groups exhibited significantly higher levels of ALP (Fig 5b). Our results suggest that TMSC-GHH treatment induced bone formation regardless of injection frequency, and this effect was more robust than that induced by estrogen treatment.

To determine the presence of unwanted disturbances in blood calcium levels, serum total calcium was measured at the end of the experiment. None of the mice in our experiment showed any critical deviation from the normal calcium level (Fig 5c).

### Postmortem organ examination of OVX mice

**Fig 6:**
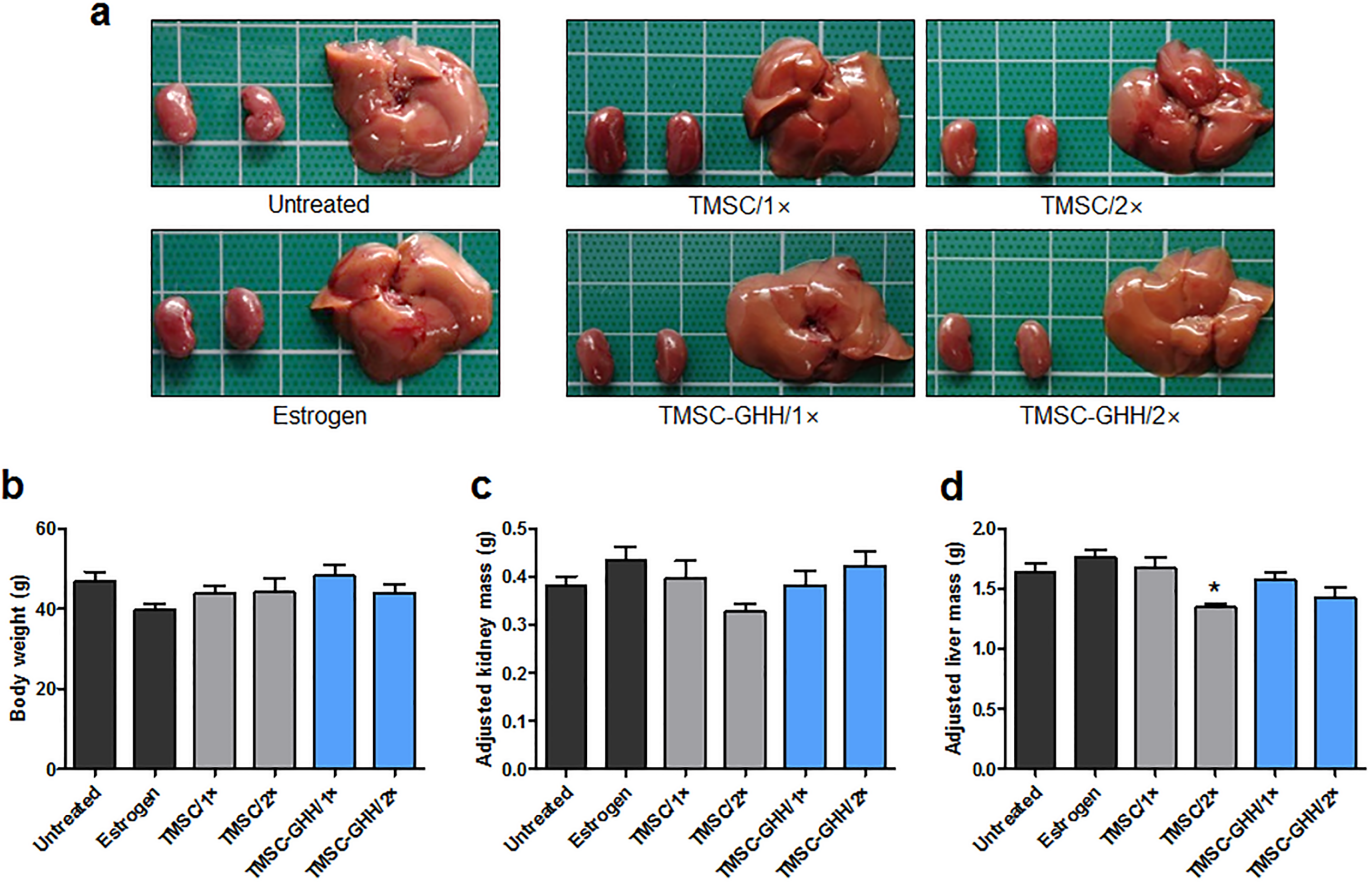
Postmortem kidney and liver samples from OVX mice. (a) Macroscopic morphology of representative postmortem kidneys and liver obtained from each group at necropsy. (b) Mean body weight of each group 3 months after first treatment. (c) Mean body weight-adjusted kidney mass values at 3 months after treatment. (d) Mean body weight-adjusted liver mass values at 3 months after first treatment. Statistical significance is marked as *P < 0.05.

At the end of the experiment, all mice were sacrificed and their internal organs were examined. Macroscopically, postmortem livers and kidneys did not display any morphological defect characteristic of pathologic conditions, such as hepato/nephrotoxicity or hepatitis/nephritis (*e*.*g*., nodules, enlargement, swelling, discoloration, or any other deviation from normal characteristics) (Fig 6a). The organs of the different treatment groups were similar in appearance.

Bilateral kidneys and liver from each mouse were weighed, the measurements were adjusted by body weight, and the values were examined for any deviation that could indicate toxic effects on these organs [41]. At the experimental endpoint, the OVX mice of all experimental and control groups were largely similar to one another in terms of body weight (Fig 6b), kidney mass (Fig 6c), and liver mass (Fig 6d).

### TMSC-GHH reduces visceral fat

**Fig 7:**
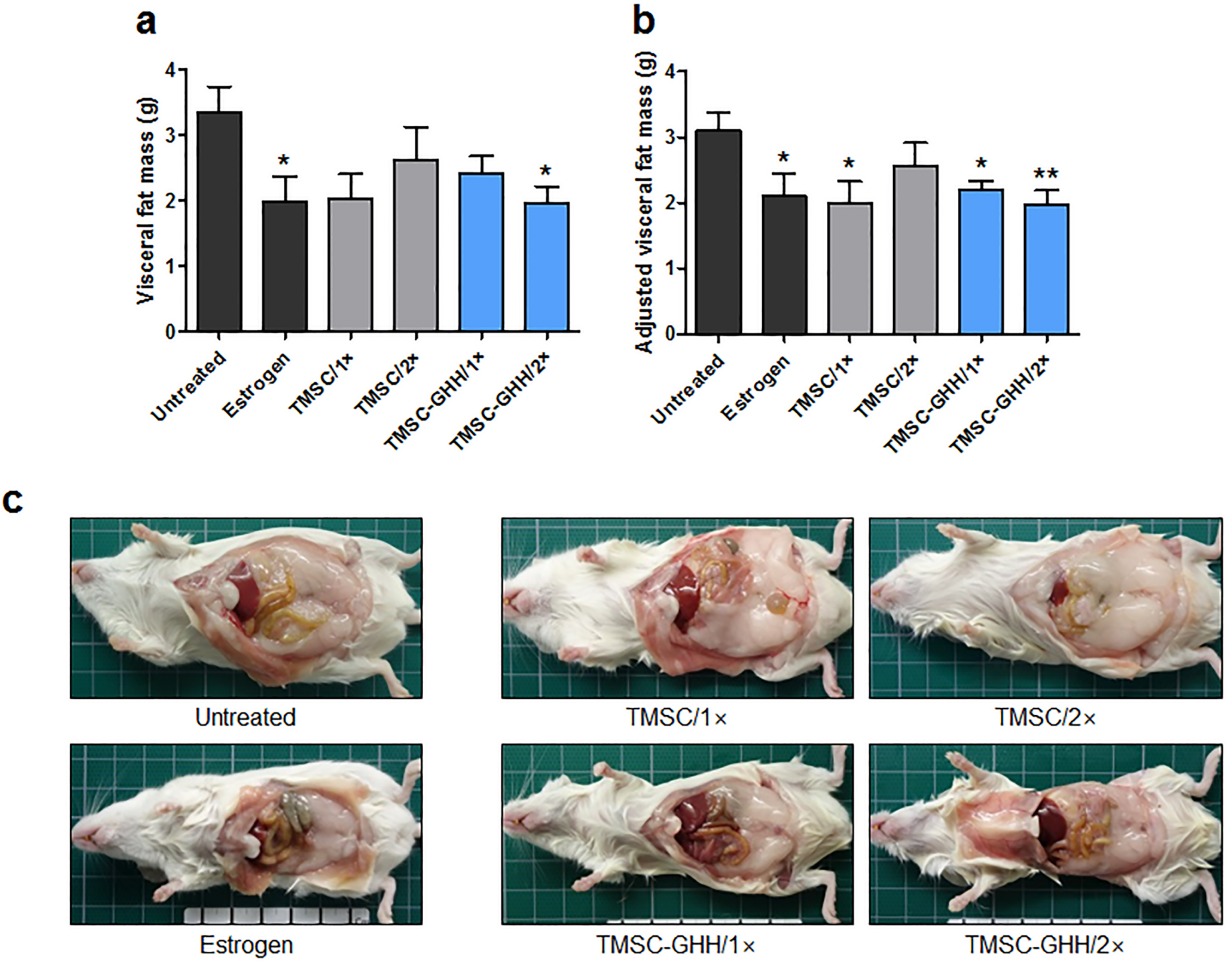
Body weights and visceral fat masses of OVX mice at the experimental endpoint. Visceral fat pads from periovarian and parametrial regions were collected and weighed at necropsy, which took place 3 months after treatment. (a) Mean visceral fat mass of each group. (b) Mean body weight-adjusted visceral fat mass values. (c) Representative macroscopic observations from each group of internal organs and fat pads after the peritoneum was cut open at necropsy. Statistical significance is marked as *P < 0.05 and **P < 0.01.

Visceral fat was recently illustrated to release proinflammatory cytokines that can exacerbate osteoporosis [42]. To elucidate the effects of our TMSC therapies on the visceral fat mass, visceral fat was collected from each mouse at necropsy and weighed. Estrogen-treated mice had a significantly lower visceral fat mass compared to untreated mice (Fig 7a). Similar to this positive control, TMSC-GHH/2× mice showed significantly reduced visceral fat. In contrast, TMSC alone did not significantly reduce visceral fat. We next adjusted the visceral fat mass values by the mean body weights to yield adjusted visceral fat mass values (Fig 7b). After this adjustment, both TMSC-GHH treatment groups still had significantly lowered visceral fat, and the visceral fat reduction of the TMSC/1× group reached statistical significance. Similar patterns were observed upon gross morphological examination of the abdominal cavities at necropsy (Fig 7c).

## Discussion

As evidence continues to emerge regarding the relationship between decreasing MSC function and osteoporosis [20, 23,43], MSC therapy is becoming an increasingly attractive alternative or additive for the current osteoporosis treatments. Research groups previously showed that MSC transplantation has therapeutic potential in age-related and postmenopausal osteoporosis animal models [24]. However, systemically delivered MSC interfere with the circulation [25] and the immune functions [26]. Here, we attempted to expand on the prior studies by employing safe and convenient subcutaneous injection [44]. We report that subcutaneous injection of TMSC alleviated postmenopausal osteoporosis, but only when the cells were embedded in the hydrogel scaffold, GHH.

The size difference of GHH at the time of injection (Fig 3c) and at the endpoint (Fig 3d) indicates GHH degradation over 3 months. GHH is known to degrade only in the presence of proteolytic enzymes, and the process of degradation does not stimulate inflammatory responses to further advocate its biocompatibility [35,45]. Seeded TMSC are expected to stay attached to GHH and washed away as GHH undergoes degradation, in the same way with growth factors delivered via gelatin hydrogel carriers; those factors were previously shown to be released only with degrading gelatin, following the initial burst release [45].

As expected, subcutaneous injection of TMSC alone failed to reverse osteoporosis. Similar results were obtained for subcutaneous TMSC treatment of hypoparathyroidism, which showed therapeutic efficacy only with a scaffold use [15,17]. TMSC injected alone are likely to be diffused to surrounding area and lost to be nonfunctional, and GHH scaffold is essentially required for proper functioning of subcutaneously injected TMSC in this context. The major function of gelatin-based hydrogel in this study was to work as scaffold to support the embedded TMSC, so that TMSC can influence bone formation and osteoporosis treatment via a paracrine manner.

Although TMSC-GHH was injected in the dorsum area (Fig 2b), bone regeneration was observed in the femoral head trabeculae away from the injection site (Fig 4a). Recovery is likely to be due to paracrine effects of TMSC-GHH. Recently revealed mechanisms of MSC functions include release of secretory factors [36], exosomes and microvesicles, extending from the traditional role of replacing damaged cells [46,47]. TMSC are good modulators of tissue microenvironment, as its transcriptome include more extracellular, protein-binding proteins, and immunomodulatory molecules compared to other MSC [8]. Moreover, we previously reported that conditioned medium of TMSC is therapeutic toward senile osteoporosis [20]. Therefore, regenerative efficacy of TMSC-GHH is conceivably through its secretome.

As previously mentioned, the strength of GHH, along with various other parameters, can be easily controlled. For example, we previously manipulated the H_2_O_2_ concentration in this *in situ* forming gelatin hydrogel to obtain a stiffness of 1.1 kPa, and showed that this improved the wound-healing function of embedded fibroblasts, better than 6.2 kPa hydrogel [27]. Here, we obtained better recovery of bone loss during osteoporosis, compared to that achieved by estrogen treatment, when TMSC were embedded in GHH scaffold with a stiffness of 4.4 kPa (Fig 4a and 4b).

For all tested treatment groups, serum OCN concentration was generally lower and serum ALP concentration was generally higher (especially in the TMSC-GHH groups) compared to those of controls (Fig 5a and 5b). The tendency of serum OCN to decrease following treatment appears to suggest recovery from the heightened serum OCN level caused by OVX. It has been demonstrated that increased serum OCN levels may be used as an indication of osteoporosis [48], and that these levels are decreased by treatment [37,49]. Serum ALP is a well-known marker of bone formation, and the efficacy of osteoporosis treatment can be determined by its increase [37,50]. Estrogen-treated MSC were previously reported to display increased and decreased expression levels of the mRNA for ALP and OCN, respectively [51], which matches the pattern observed in our present treatment groups.

OVX mice treated with TMSC-GHH showed a more favorable serum marker pattern than even the positive control (estrogen-treated) mice; serum OCN was lower in TMSC-GHH/2× mice than in any other group, and serum ALP was significantly increased in both TMSC-GHH groups. However, our microCT analysis showed that estrogen-treated mice exhibited more filling of femoral head trabeculae than was observed for the TMSC-GHH-treated groups. This discrepancy may relate to the experimental endpoint chosen for the present study, and different results might have been obtained over a longer time course. Additional work is warranted to identify the duration required for the maximal therapeutic efficacy of TMSC-GHH in OVX mice.

Kidney and liver weights as well as gross morphology stayed constant in TMSC-GHH-treated mice (Fig 6). These observations indicate that the TMSC and GHH were apparently well-tolerated and biocompatible. This was consistent with a previous study, where subcutaneously implanted gelatin hydrogel was well-integrated without fibrous capsule formation or inflammatory reactions *in vivo* [52].

Our findings indicate that the therapeutic application of TMSC, especially TMSC-embedded GHH, can effectively reduce visceral fat (Fig 7). Recent studies suggest that visceral fat is especially harmful for bone health [53], and the shift of resident MSC from an osteogenic tendency to an adipogenic tendency reportedly contributes to postmenopausal osteoporosis [54]. In our recent study of the therapeutic efficacy of TMSC or TMSC-conditioned medium against senile osteoporosis, we showed that the tested injections reduced marrow adipose tissue [20]. Further studies are needed to examine whether the reduction of visceral fat mass observed under our experimental conditions could contribute to preserving bone strength.

Taken altogether, the present results indicate that the injection frequency had little influence on the therapeutic efficacy. The serum OCN level of TMSC-GHH/2× mice was significantly lower than that of TMSC-GHH/1× mice (Fig 5a), but the other tested parameters, including BMD (Fig 4b), serum ALP (Fig 5b) and adjusted visceral fat mass (Fig 7b), did not differ between the dosage schedules. These observations indicate that a single injection was sufficient to confer the therapeutic benefits observed in the present study. In the future, it could be useful to examine whether the tested 3-month period is the most appropriate for maximal efficacy and/or whether more frequent injections might help or stall the therapeutic benefits.

## Conclusion

**Fig 8:**
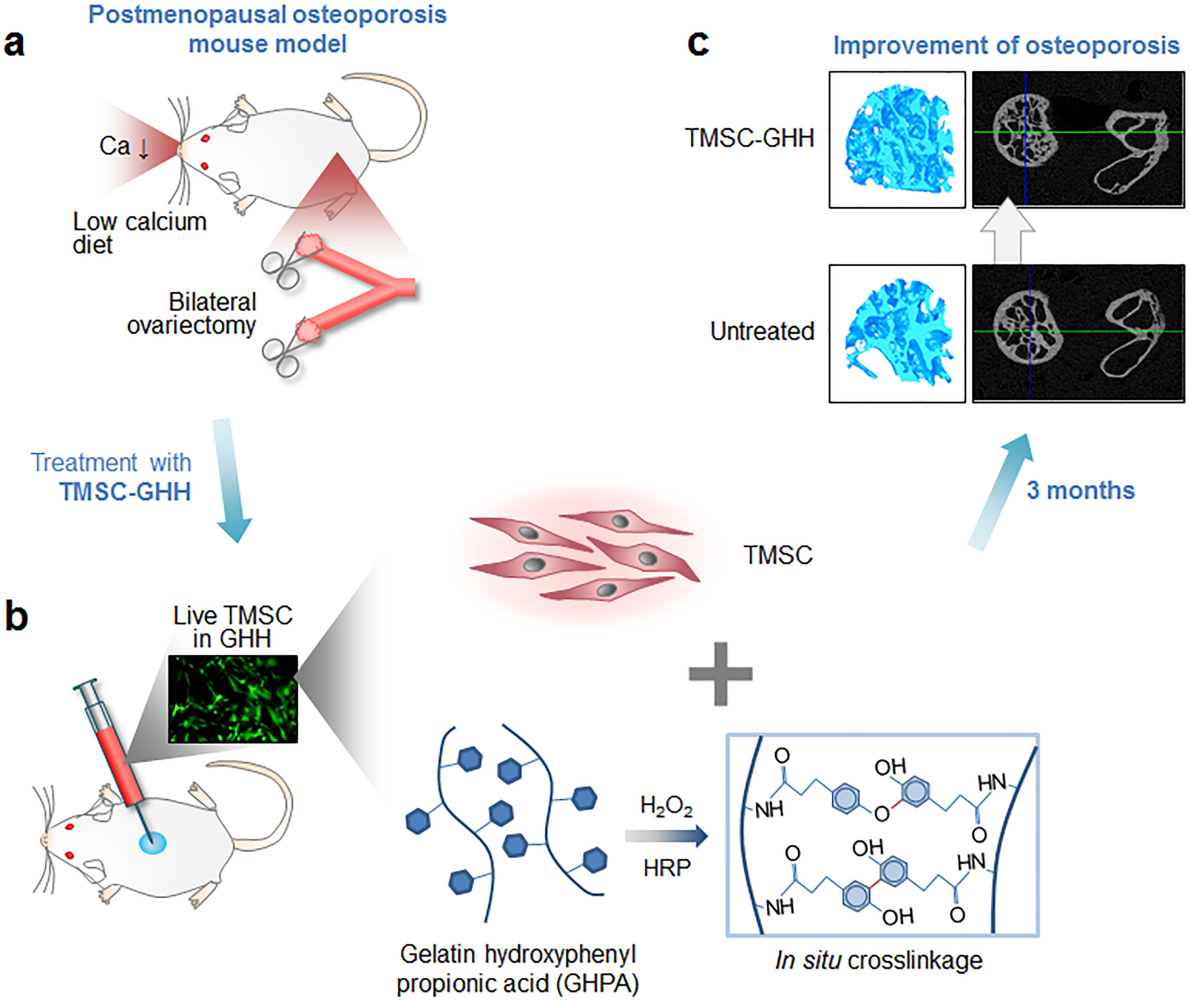
Schematic representation of bone regeneration by TMSC-embedded GHH. (a) Bilateral OVX osteoporosis mouse model was used for investigating bone formation effect of TMSC-embedded GHH. (b) GHPA polymers were enzymatically *in situ* crosslinked and formed GHH, and TMSC-embedded GHH was subcutaneously injected into OVX mice. (c) Improved microCT results were observed in TMSC-GHH-treated mice 3 months after the initial treatment.

The present study showed the potential of TMSC-GHH treatment for osteoporosis. TMSC-GHH improved serum markers related to osteoporosis as well as trabecular structures and bone parameters of the femoral head (Fig 8).

Additional work will be required to further optimize the experimental conditions (*e*.*g*., the number of cells to be implanted and the injection route) in the hope of boosting the therapeutic efficacy of our system, as well as to examine the molecular mechanism underlying the therapeutic effects of TMSC-GHH.
